# Acupuncture as an early treatment for idiopathic sudden sensorineural hearing loss (ISSNHL) patients with flat or high-frequency drop audiograms: study protocol for a randomized controlled trial

**DOI:** 10.1186/s13063-018-2737-x

**Published:** 2018-07-04

**Authors:** Kai Shang, Xin Ma, Hui-Lin Liu, Yuan-Yuan Jing, Lin Zeng, Nan Li, De-An Zhou, Jia Wei, Chen Zhang

**Affiliations:** 1 Acupuncture and Moxibustion Department, Beijing Hospital of Traditional Chinese Medicine affiliated with Capital Medical University, No.23, Mei shu guan hou jie Road, Beijing, 100010 China; 2 Otorhinolaryngology Department, Peking University People’s Hospital, No.11, Xi zhi men nan da jie Road, Beijing, 100044 China; 3 Research Center of Clinical Epidemiology, Peking University Third Hospital, No.49, Hua yuan bei lu Road, Beijing, 100191 China

**Keywords:** Acupuncture, ISSNHL, Flat, High frequency, Study protocol, Randomized controlled trial

## Abstract

**Background:**

Idiopathic sudden sensorineural hearing loss (ISSNHL) is a common form of deafness. Acupuncture has been used as a salvage therapy for ISSNHL in China since 200 BCE. However, the efficacy of acupuncture has not been confirmed in strictly controlled trials. We designed a randomized controlled clinical trial to evaluate the efficacy and long-term effects of acupuncture in patients with early ISSNHL.

**Methods/Design:**

In this randomized controlled clinical trial, we will enroll 124 participants with ISSNHL diagnosed 2 to 4 weeks prior to enrollment, who have shown little hearing improvement after routine Western medical treatment (i.e., corticosteroids). 62 of these participants will have flat audiogram and the other 62 will have a high-frequency drop audiogram; they will all take Methycobal while half of the flat type and half of the high-frequency drop type will also receive acupuncture treatments for 4 weeks in a four-group design. The primary outcome measure will be the effective rate of hearing improvement (defined as the proportion of patients with at least 15-dB improvement in the hearing loss frequency band). The secondary outcome will measure the improvements in Pure Tone Average, Word Recognition Score, and Tinnitus Handicap Inventory. The assessments of the participants will be made at baseline, after treatment (week 4), and at follow-up (week 28).

**Discussion:**

This study aims to explore the efficacy and long-term effects of acupuncture in patients with ISSNHL. This study will be a randomized controlled trial with strict methodology and few design deficits. If our study yields positive results, acupuncture could be recommended as a salvage therapy for patients with ISSNHL.

**Trial registration:**

Chinese Clinical Trial Registry, ChiCTR-ICR-15006787. Registered on 12 July 2015.

**Electronic supplementary material:**

The online version of this article (10.1186/s13063-018-2737-x) contains supplementary material, which is available to authorized users.

## Background

Idiopathic sudden sensorineural hearing loss (ISSNHL) is a common type of deafness in adults and is defined as a sudden hearing loss (≥30 decibels Hearing Level, or dB HL) that develops in less than 72 h and affects three consecutive frequencies [1]. Epidemiological studies from different regions, such as the United States, Sweden, Japan, and Taiwan, suggest that ISSNHL has an annual incidence rate of 5 to 30 out of 100,000 [2–5], another study in Germany confirmed an annual incidence rate of 160 out of 100,000 [6], and the rates are increasing [7]. Concomitant symptoms include a full or blocked ear, vertigo, and tinnitus [2]. Tinnitus can cause extreme anxiety and depression [2]. In China, it was reported that tinnitus was experienced by 70% to 100% of patients with ISSNHL [8]. About one third to two thirds of patients with ISSNHL recover within a couple of weeks [2, 9–11], while after 2 weeks patients would be unlikely to show much recovery [2, 10, 12, 13]. Corticosteroids have been suggested for treatment of ISSNHL within the first 2 weeks [2, 14–16]. However, at present, generally accepted therapies for patients with longer-term ISSNHL are still lacking.

The treatment and prognosis of ISSNHL are closely related to the patient’s audiogram type [17]. According to the German guidelines, ISSNHL has six types [18]: (1) hearing loss at low frequencies, in the range of 250–500 Hz, which has the best prognosis; (2) hearing loss at middle frequencies, around 1000 Hz; (3) hearing loss at high frequencies, above 4000 Hz, which has a poor prognosis; (4) flat type, hearing loss at all frequencies, which also has a poor prognosis; (5) complete/profound deafness, extreme hearing loss at every frequency, which has the worst prognosis; and (6) others unspecified. A multi-center randomized controlled trial (RCT) for a pharmacological treatment in China [19] obtained type-differentiated prognosis data similar to those described in the German guidelines. Some studies showed that ISSNHL with low-frequency drop audiograms has the best prognosis and responds well to treatments [18, 20, 21], complete/profound deafness is difficult to treat [18], and patients with middle-frequency drop audiograms are rare [18]; therefore, ISSNHL patients with flat or high-frequency drop audiograms are more suitable for research than other types. Acupuncture therapy is a significant component of traditional Chinese medicine (TCM). In TCM theory, it is believed that ISSNHL is caused by the Yin-Yang imbalance of internal organs. Acupuncture is considered a useful treatment that brings about balance by stimulating acupoints to activate channels and improve and regulate the functions of Zang-Fu organs, Qi, and Blood according to *Huang Di Nei Jing* (a famous work of ancient TCM literature). Since ancient times in China, there have been reports of acupuncture being used to treat ISSNHL symptoms [22]. Recent studies have suggested that acupuncture may have beneficial effects on ISSNHL [23, 24], but evidence of acupuncture studies that have employed audiograms is limited. Furthermore, prior acupuncture studies did not account for the amount of time elapsed since ISSNHL onset, which is an important prognosis factor that may influence treatment outcomes. Therefore, this randomized controlled clinical trial was designed to evaluate the efficacy and long-term effects of acupuncture as a salvage therapy for patients who have not recovered 2 to 4 weeks after the onset of ISSNHL.

## Methods/Design

### Objective

This RCT will examine the efficacy of acupuncture as an early treatment for ISSNHL patients with flat or high-frequency drop audiograms.

### Study design

**Fig. 1:**
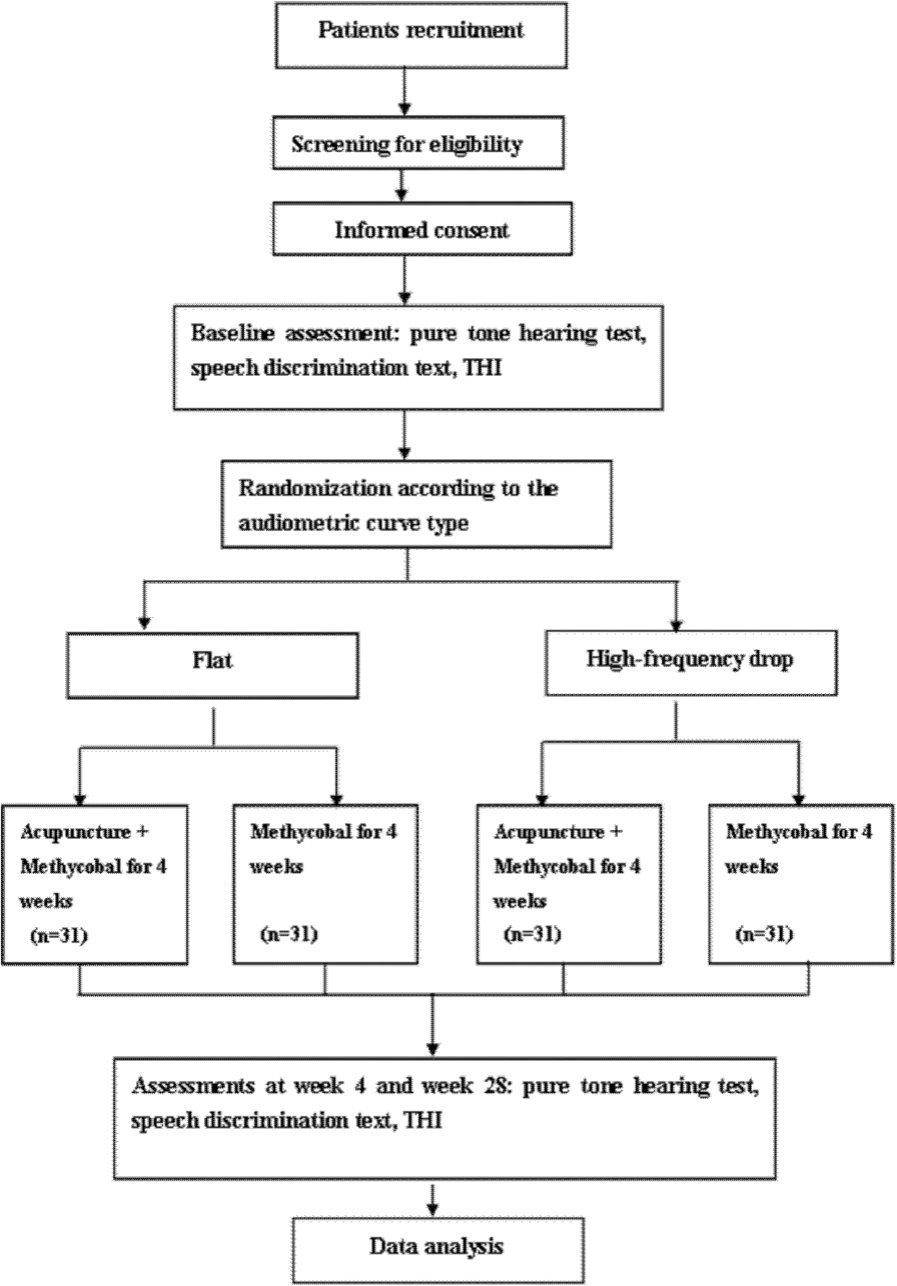
Trial flow chart. Abbreviation: *THI* Tinnitus Handicap Inventory

**Fig. 2:**
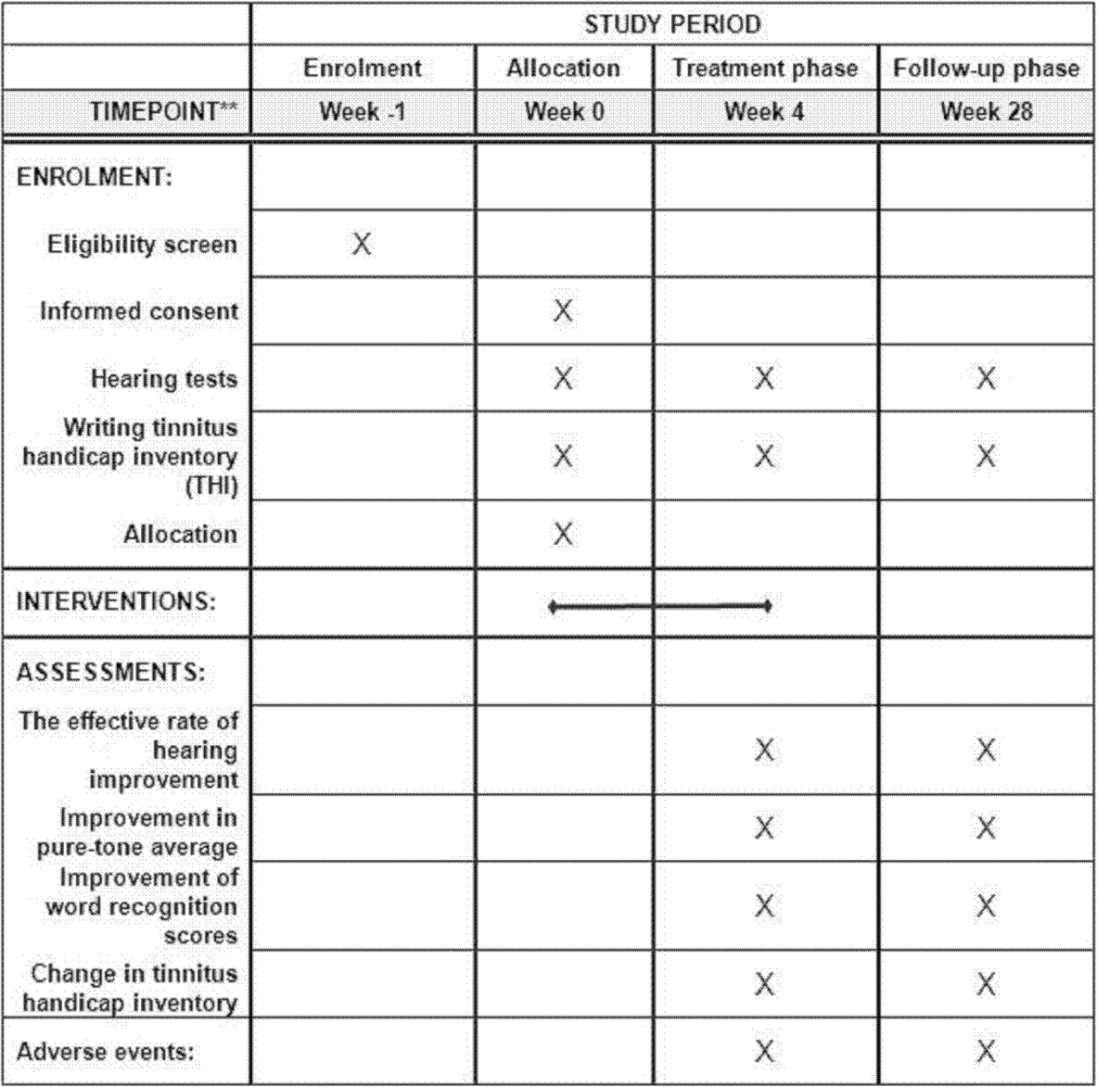
Chart of trial processes. **refers to “Specific timepoints in this study”

This RCT will be undertaken at two sites: Beijing Traditional Chinese Medicine Hospital Affiliated with Capital Medical University and Peking University People’s Hospital. In this study, we will compare the efficacy of Methycobal with or without acupuncture treatment in ISSNHL patients with flat or high-frequency drop audiograms (Figs. 1 and 2). Patient recruitment, hearing testing, baseline assessments, and follow-up evaluations will be conducted in the Department of Otorhinolaryngology at Peking University People’s Hospital. Acupuncture treatments on the study participants will be provided in the Acupuncture and Moxibustion Department of Beijing Traditional Chinese Medicine Hospital Affiliated with Capital Medical University. Both the participants and the acupuncturist will be aware of the treatment groups, but investigators performing assessments will be blinded. The acupuncture treatment will consist of 12 sessions over a period of 4 weeks. The participants will be assessed at baseline, post-treatment (week 4), and follow-up (week 28). This trial will be performed in accordance with the principles of the Declaration of Helsinki (Version Fortaleza 2013). The study protocol has been approved by the Research Ethics Committee of Beijing Traditional Chinese Medicine Hospital Affiliated with Capital Medical University (reference 2014BL-063-02). All participants will sign an informed consent form. The trial will be conducted from September 2018 to June 2020.

To ensure that the treatments and hearing tests are reliable, two hospitals will participate in this trial: (1) Beijing Traditional Chinese Medicine Hospital Affiliated with Capital Medical University and (2) Peking University People’s Hospital. The Department of Otorhinolaryngology at Peking University People’s Hospital has a sufficient number of patients with acute ISSNHL for the study group and provides standard Western medical treatments. An ear-nose-and-throat doctor will be in charge of patient recruitment, inclusion, hearing tests, and follow-up. The acupuncture treatments will be carried out by highly experienced acupuncturists with a minimum of 20 years of acupuncture experience at Beijing Traditional Chinese Medicine Hospital. The planned acupuncture paradigm was based on the clinical experience of the nationally renowned veteran doctor of TCM, Zhou De’an, who has been using acupuncture to treat hearing loss for more than two decades. Our team also contains a research methodologist to ensure scientific integrity and feasibility.

### Randomization

Randomization will be accomplished by using SAS 9.1 software (SAS Institute Inc., Cary, NC, USA) and will be carried out by the Research Center of Clinical Epidemiology Affiliated with Peking University Third Hospital. For each of the two ISSNHL types, random allocation sequences will be generated, and opaque sealed envelopes will be created by computer. For both types, the ratio of patients in the treatment group and control group will be 1:1. The envelopes will be attached to one another in numerical sequence, and each envelope will be opened in sequence when a new patient is registered in the trial.

### Participants

#### Inclusion criteria

1. Ages between 18 and 70;
2. Diagnosis of ISSNHL [1, 25], defined as sudden hearing loss (≥30 dB) in less than 72 h affecting three consecutive frequencies;
3. ISSNHL patients with flat or high-frequency drop audiograms according to pure tone audiometry;
4. ISSNHL began 2 to 4 weeks prior;
5. Hearing improvement of less than 50% since ISSNHL began.

#### Exclusion criteria

1. Hearing loss from other causes, including congenital deafness, conductive deafness, presbycusis, large vestibular aqueduct syndrome, Meniere’s disease, tumor, or deafness due to systemic genetic diseases;
2. Presence of serious diseases such as insufficiency of heart, liver, and kidney organs or coagulation disorders;
3. Pregnancy or lactating process in females.

### Intervention

Participants in the treatment groups will receive oral Methycobal (0.5 mg three times a day) and verum acupuncture for 4 weeks, while those in the control groups will receive similar Methycobal treatment but without acupuncture. The acupuncture treatment will consist of three sessions per week at the following acupuncture points: Baihui (DU20), Shenting (DU24), Ermen (SJ21), Tinggong (SI19), Tinghui (GB2), Jiaosun (SJ20), Yifeng (SJ17), Waiguan (SJ5), Zhongzhu (SJ3), Zhubin (KI9), Qiuxu (GB40), Taichong (LR3), and Zulinqi (GB41). For the acupuncture treatments, only sterilized single-use needles (Hwato Needles, Suzhou Medical Appliance Factory, Suzhou New District, China) will be used. For limb and abdomen acupoints, 30-gauge needles (0.3 mm in diameter, 40 mm long) will be inserted to a depth of 10 to 15 mm. For head acupoints, 32-gauge needles (0.25 mm in diameter, 25 mm long) will be inserted to a depth of 3 mm. In addition, a special acupuncture manipulation will be used. The acupuncturist will insert a 30-gauge needle (0.3 mm in diameter, 40 mm long) through three acupoints, from Ermen (SJ21) through Tinggong (SI19) to Tinghui (GB2), at an angle of 15° while the patient has his or her mouth open. All needles will be manually manipulated by rotation methods to produce the characteristic sensation known as De Qi. De Qi is a special sensation during acupuncture treatment. When the needle gets to the acupoint, patients always have muscle tenseness around the needle with some feelings such as numbness, distension, soreness, or heaviness. Once De Qi is achieved, needles will be kept *in situ* for 30 min without further manipulations.

### Outcome measures

Audiometry (pure tone hearing test), Word Recognition Score (WRS), and Tinnitus Handicap Inventory (THI) will be used to assess the participants before and after treatment (at weeks 4 and 28). The primary outcome measure will be the effectiveness of hearing improvement, defined as the proportion of patients with an improvement of at least 15 dB in their hearing loss frequency band [25]. For hearing improvement, complete recovery will be defined as hearing level equal to that of the unaffected ear or to hearing prior to ISSNHL [25]. Hearing gains of greater than 30 dB will be considered significant, gains of 15 to 30 dB will be considered effective, and gains of less than 15 dB will be considered ineffective [25]. The secondary outcome will measure the improvements in Pure Tone Average (PTA) (dB HL) [2], in WRS [2] and in THI [26].

### Blinding

Data managers and statisticians will be blinded throughout the trial. During the study, therapists and data managers will be requested not to communicate with each other about the patient groups. Blinded telephone interviewers will collect the follow-up materials to evaluate the long-term effect of acupuncture at 28 weeks after baseline.

### Sample size

Sample size was calculated on the basis of a single-arm pilot study that showed an effective rate of hearing improvement in 33% of the acupuncture-plus-Methycobal group according to the Chinese sudden hearing loss multi-center clinical study group [19]. We assumed that the effective rate of hearing improvement in the group with only Methycobal was 5%. The difference of the effective rate between the two groups was 28%. This study will be based on 0.8 power to detect a significant difference (α = 0.05, two-sided). In this case, it means that 26 participants will be required for each group, as calculated using PASS 2008. To allow for a 15% withdrawal rate, we plan to enroll 31 participants per group, amounting to a total of 124 participants.

### Patient safety

To ensure patient safety, any adverse events (described as unfavorable or unintended signs, symptoms, or diseases occurring after treatment) related to acupuncture treatment will be noted and reported by patients and practitioners during each patient visit; in addition, all vital signs and adverse events will be measured and recorded at these visits.

### Data management

Two independent researchers blinded to the group allocation will separately enter the data on an Excel spreadsheet after the completion of the case report forms (CRFs). Another independent researcher will crosscheck and compare the two datasets. If different data entry is discovered, it will be compared with the original CRFs to identify any inconsistency. All modifications will be marked on the CRFs. The raw research data will be gathered and saved on a computer. Paper files will be kept in a locked filing cabinet. Electronic documents will be stored in a password-protected computer, and access will be restricted to the principal investigator only. All research documents will be stored for at least 5 years after publication.

### Quality control

All acupuncturists and assessors will be required to undergo special training prior to the trial to guarantee consistent practices. The training program will include diagnosis, inclusion and exclusion criteria, location of the acupuncture points, acupuncture manipulation techniques, and completion of CRFs. Study participants who withdraw from the study will be recorded during the intervention and follow-up periods. This trial will be monitored by the scientific research department of Beijing Traditional Chinese Medicine Hospital. There will be periodic monitoring which will guarantee accuracy and quality throughout the study period.

### Statistical analyses

The de-identified outcome data will be analyzed by a statistician blinded to group allocations using the Statistical Package for the Social Sciences V.19.0 statistical software package (SPSS, Chicago, IL, USA). Significant levels will be set at a *P* value of less than 0.05. Data analysis of baseline characteristics and of primary and secondary outcomes will be based on the intention-to-treat principle. The chi-squared test will be conducted for proportions and independent samples. The *t* tests will be analyzed for testing the baseline differences between the two groups. A repeated-measures analysis of variance (ANOVA) will be used to compare the effect of the treatments between the two study groups. Any missing data will be replaced according to the principle of the last observation and carried forward.

## Discussion

As many as two thirds of patients with ISSNHL may recover within 2 weeks [2, 9–11]; some do not achieve any additional recovery after this time [12, 13]. Current methods to treat ISSNHL are insufficient. In our previous research, we found that 30% to 40% of patients with ISSNHL experienced hearing improvement after acupuncture treatment. However, the beneficial effects of acupuncture treatment have not been verified through an RCT. This trial will be the first RCT of acupuncture based on audiometric curves. ISSNHL with low-frequency drop audiograms has the best prognosis after routine Western medical treatment [18]; the effective rate is more than 75% [20, 21]. Conversely, complete/profound deafness has a very poor prognosis [18]. ISSNHL with middle-frequency drop audiograms is rare [18]. The first 2 weeks after the onset of ISSNHL offer the best opportunity for recovery [2, 12]. Patients who do not show any improvement within the first 2 weeks are unlikely to have much recovery afterwards [6, 10]. It has been suggested that acupuncture can be used as a salvage therapy for ISSNHL within 4 weeks after the onset of the disease. However, prior studies examining acupuncture for ISSNHL have not sufficiently addressed the period from its onset. Therefore, we chose early ISSNHL patients with flat or high-frequency drop audiograms to evaluate the efficacy of acupuncture treatment.

The most common ISSNHL assessment parameter, the effective rate of hearing improvement, will be used in this trial as the primary outcome measure. We will assess changes in PTA, WRS, and THI as the secondary outcome measures. WRS is widely used to evaluate hearing because the most commonly reported complaint in patients with hearing loss is the inability to understand speech [27]. Furthermore, American guidelines recommend the use of WRS to assess the effectiveness of treatment [2]. THI is also used to assess treatment effectiveness because more than 70% of patients with ISSNHL have tinnitus, which can cause extreme anxiety and depression [2].

Generally accepted treatments for longer-term ISSNHL are still lacking. Even though some Western medicines have been suggested, the evidence of their efficacy is still unclear. This study aims to explore the efficacy of acupuncture treatment for patients with ISSNHL on top of Western medicine treatment. In this study, participants in the control group will receive Methycobal only, without sham acupuncture, because all patients in the control group will be recruited from a Western medical hospital which does not offer acupuncture treatment and this means that these patients would not seek acupuncture treatment. In some acupuncture research, sham acupuncture was used in order to decrease the bias of subjective quantity from patients, such as pain. In this study, the primary outcome measure will be focused on the effective rate of hearing improvement, as this is not a subjective measure, so sham acupuncture is unnecessary for this study.

This study will have its own limitations and some of them include that, for ethical reasons, there will not be a treatment group that receives acupuncture only. Second, neither the acupuncturist nor the patients will be blinded to treatment. It is almost impossible to have genuine double blinding in acupuncture trials because most participants in China have basic acupuncture knowledge and acupuncturists require patient feedback during the needling process. This article presents the design and protocol of an RCT of acupuncture as an early treatment and salvage therapy for ISSNHL. Completion of this trial will clarify the efficacy of acupuncture to treat ISSNHL patients with flat or high-frequency drop audiograms.

It is believed that acupuncture can treat ISSNHL and the possible basis for this belief might be that acupuncture can promote blood flow to the ear and decrease blood viscosity [28, 29]. Another study showed that acupuncture can improve blood circulation in the ear and increase the oxygen supply in the ear, all of which are important factors in promoting auditory recovery [30]. Other studies have found that acupuncture at acupoints around the ear may reduce blood viscosity, regulate the inflammatory response, improve lymph circulation, and enhance the excitability and conductivity of the auditory nerve [31, 32]. The exact mechanisms that explain acupuncture effects on ISSNHL have not yet been resolved; hence, further detailed research and discussion are needed in the future Additional file 1.

## Trial status

Recruitment will start in September 2018 and will be completed by the end of June 2020.

## Additional file

Additional file 1: SPIRIT 2013 Checklist: Recommended items to address in a clinical trial protocol and related documents*. (DOC 136 kb)

## Abbreviations

CRF: Case report form
dB HL: Decibels Hearing Level
ISSNHL: Idiopathic sudden sensorineural hearing loss
PTA: Pure Tone Average
RCT: Randomized controlled trial
TCM: Traditional Chinese medicine
THI: Tinnitus Handicap Inventory
WRS: Word Recognition Score
