# Supplementary material for: Acupuncture as an early treatment for idiopathic sudden sensorineural hearing loss (ISSNHL) patients with flat or high-frequency drop audiograms: study protocol for a randomized controlled trial
Source: Trials. 2018 Jul 4;19:356. doi: 10.1186/s13063-018-2737-x (PMC6032598; doi:10.1186/s13063-018-2737-x)
Supplement: Supplementary file 1 — SPIRIT 2013 Checklist: Recommended items to address in a clinical trial protocol and related documents*. (DOC 136 kb) [file 13063_2018_2737_MOESM1_ESM.doc]

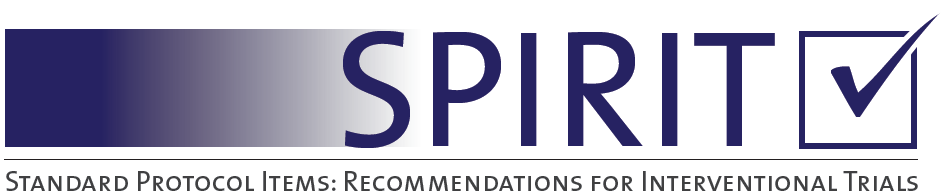


SPIRIT 2013 Checklist: Recommended items to address in a clinical trial protocol and related documents*

| Section/item | Item No | Description | Addressed on page number |
| --- | --- | --- | --- |
| **Administrative information** | | |  |
| Title | 1 | Acupuncture as an early treatment for idiopathic sudden sensorineural hearing loss (ISSNHL) of flat type or high-frequency drop: protocol of a randomized controlled trial | _____1________ |
| Trial registration | 2a | Chinese Clinical Trial Registry: ChiCTR-ICR-15006787 | ______2_______ |
|  | 2b | All items from the World Health Organization Trial Registration Data Set | ______X_______ |
| Protocol version | 3 | 2017-02-20 Version 1.0 | ______X_______ |
| Funding | 4 | Beijing Municipal Science & Technology Commission of China (Z141107002514069), Beijing Municipal Commission of Health and Family Plaining (首发2014-2-2232), Beijing Municipal Administration of Hospitals Clinical Medicine Development of Special Funding Support (ZYLX201412) and National Basic Research Program of China under Grant (2014CB543203) | ______14______ |
| Roles and responsibilities | 5a | Kai Shang, Hui-Lin Liu, De-An Zhou, Jia Wei and Chen Zhang are from Acupuncture and Moxibustion Department, Beijing Hospital of Traditional Chinese Medicine affiliated to Capital Medical University, Beijing, China. Xin Ma and Yuan-Yuan Jing are from Otorhinolaryngology Department, Peking University People’s Hospital, Beijing, China. Lin Zeng and Nan Li are from Research Center of Clinical Epidemiology, Peking University Third Hospital, Beijing, China.  H-L L, X M, Y-Y J, L Z, N L and D-A Z, contributed to the design of the study, drafting and editing of the manuscript. H-L L and K S wrote the first manuscript for this trial. X M and L Z edited the final manuscript. J W and C Z plan for the statistical analysis. H-L L will conduct the acupuncture operation. All authors read and approved the final manuscript. | _____1, 15____ |
|  | 5b | Beijing Municipal Science & Technology Commission of China (Telephone number: 0086 010 66153395) | ______14_______ |
|  | 5c | Beijing Municipal Science & Technology Commission of China is the sponsor and will not affect the result of this trial. | ______14_______ |
|  | 5d | This trial will be monitored by the scientific research department of Beijing TCM Hospital. | ______11_______ |
| Introduction |  |  |  |
| Background and rationale | 6a | Idiopathic sudden sensorineural hearing loss (ISSNHL) is a common form of deafness. Acupuncture has been used as a salvage therapy for ISSNHL. However, the efficacy of acupuncture has not been confirmed in strictly controlled trials. We designed a randomized controlled clinical trial to evaluate the efficacy and long-term effects of acupuncture in early ISSNHL patients. | ______3_______ |
|  | 6b | Intervention for control group will be only Methycobal, because these patients with ISSNHL do not recover within 2 weeks, Methycobal is the routine medicine treatment. Control group will not receive sham acupuncture because all patients are come from western medicine hospital, they do not expect acupuncture, so there will not be selection bias. The other reason is that most participants in China have basic acupuncture knowledge. | ____13,14_____ |
| Objectives | 7 | This trial will examine the efficacy of acupuncture as an early treatment for flat type and high-frequency drop ISSNHL. | _______4______ |
| Trial design | 8 | a randomized controlled trial | _______4______ |
| Methods: Participants, interventions, and outcomes | | |  |
| Study setting | 9 | Two academic hospitals in China. | ______4_______ |
| Eligibility criteria | 10 | Inclusion criteria  1. Age in the range of 18–70 years；  2. Diagnosis of ISSNHL, defined as sudden hearing loss (≥30 dB) over 72 hours affecting three consecutive frequencies;  3. ISSNHL of the flat type or high-frequency drop, according to pure tone audiometry;  4. ISSNHL onset 2–4 weeks prior;  5. Hearing improvement less than 50% since ISSNHL onset.    Exclusion criteria  Hearing loss from other causes, including congenital deafness, conductive deafness, presbycusis, large vestibular aqueduct syndrome, Meniere's disease, tumor, or deafness due to systemic genetic diseases; 2. Presence of serious diseases such as heart, liver, kidney organs insufficiency or coagulation disorders;  3. Currently pregnant or lactating. | ______8_______ |
| Interventions | 11a | Participants in the treatment group will receive oral Methycobal (0.5 mg three times a day) and verum acupuncture for 4 weeks, while those in the control group will receive the same Methycobal treatment without acupuncture. The acupuncture treatment will consist of three sessions per week at the following acupuncture points: Baihui (DU20), Shenting (DU24), Ermen (SJ21), Tinggong (SI19), Tinghui (GB2), Jiaosun (SJ20), Yifeng (SJ17), Waiguan (SJ5), Zhongzhu (SJ3), Zhubin (KI9), Qiuxu (GB40), Taichong (LR3), and Zulinqi (GB41). Sterile, single use needles (Hwato Needles, Suzhou, China) will be used. For limb and abdomen acupoints, 30-gauge needles (0.3 mm in diameter, 40 mm long) will be inserted to a depth of 10–15 mm. For head acupoints, 32-gauge needles (0.25 mm in diameter, 25 mm long) will be inserted to a depth of 3 mm. Additionally, a special acupuncture manipulation will be used. Acupuncturist will ask the patient to open his or her mouth and then insert a 30-gauge needle (0.3 mm in diameter, 40 mm-long) through three acupoints, from Ermen (SJ21) through Tinggong (SI19) to Tinghui (GB2), at an angle of 15°. All needles will be manually manipulated by rotation methods to produce the characteristic sensation known as De Qi. This is experienced as a tenseness around the needle by the practitioner and numbness, distension, soreness, and heaviness by the patient. Once De Qi is achieved, needles will be kept in situ for 30 minutes without stimulation. | _____8,9______ |
|  | 11b | When participants require to quit or have any adverse events. | ______X_______ |
|  | 11c | All participants will receive free treatment and hearing tests during the trial. | ______X_______ |
|  | 11d | Other interventions are prohibited during the trial. | ______X_______ |
| Outcomes | 12 | Audiometery (pure tone hearing test), word recognition score, and tinnitus handicap inventory (THI) will be assessed at baseline and after treatment (4 and 28 weeks). The two primary outcome measures will be: (1) the effective rate of hearing improvement, defined as the proportion of patients with an improvement of at least 15 dB in their hearing loss frequency band; and (2) improvement in pure-tone average (PTA; dB HL). For hearing improvement, complete recovery will be defined as hearing level equal to that of the unaffected ear or to hearing prior to ISSNHL onset. Hearing gains greater than 30 dB will be considered marked, gains of 15–30 dB will be considered effective, and gains of less than 15 dB will be considered ineffective. Secondary outcome measures will be: improvement of word recognition scores (WRS) and change in tinnitus handicap inventory (THI). | _____9,10_____ |
| Participant timeline | 13 |  | ______12_______ |
| Sample size | 14 | Sample size was calculated based on a one-armed pilot study that showed an effective rate of hearing improvement of 33% in acupuncture plus Methycobal group . According to the Chinese sudden hearing loss multi-center clinical study group [19]. We assumed the effective rate of hearing improvement in Methycobal only group was 5%. The difference of effective rate between two groups was 28%. Based on 0.8 power to detect a significant difference (α = 0.05, two-sided), 26 participants will be required for each group, as calculated using PASS 2008. To allow for a 15% withdrawal rate, we plan to enroll 31 per group for a total of 124 participants. | ______7,8______ |
| Recruitment | 15 | Recruitment will be conducted in the Department of Otorhinolaryngology at Peking University People’s Hospital. It has sufficient patients with early ISSNHL for the study. | ______4_______ |
| **Methods: Assignment of interventions (for controlled trials)** | | |  |
| Allocation: |  |  |  |
| Sequence generation | 16a | Randomization will be accomplished using SAS 9.1 software (SAS Institute Inc., USA), which will be written by the Research Center of Clinical Epidemiology Affiliated to Peking University Third Hospital. | ______7_______ |
| Allocation concealment mechanism | 16b | Random allocation sequences will be generated, and opaque sealed envelopes will be created by computer. | ______7_______ |
| Implementation | 16c | Peking University Third Hospital will generate the allocation sequence, Peking University People’s Hospital will enrol participants, and Beijing Hospital of Traditional Chinese Medicine will assign participants to interventions. | _____4,5______ |
| Blinding (masking) | 17a | Outcome assessors and data analysts will be blinded. | ______7______ |
|  | 17b | Trial participants will not be blinded. | ______7______ |
| **Methods: Data collection, management, and analysis** | | |  |
| Data collection methods | 18a | Before recruitment, the whole research group, including therapists, data administrators and outcome assessors, will participate in a training seminar about research contents and data management. Baseline characteristics of participants will be recorded on CRFs by a research assistant. Data collection will be conducted by a researcher who remains blinded to the group allocation at the baseline, 4 and 28 weeks from the baseline. | ______10_____ |
|  | 18b | Dropouts and withdrawals from the study will be recorded through the intervention and follow-up periods. | ______11_____ |
| Data management | 19 | Two independent researchers blinded to the group allocation will enter the data on an Excel spreadsheet after the completion of the CRFs separately. Another independent researcher will compare the two datasets for check-up. If different data entry is discovered, data will be compared with the original CRFs to verify inconsistency. All modification will be marked on the CRFs. Research data will be gathered and saved. Paper files will be kept in a locked filing cabinet. Electronic documents will be stored in a password protected computer, with access restricted to the principal investigator. All research documents will be preserved for at least 5 years after publication. | ______10_____ |
| Statistical methods | 20a | It will be conducted by statisticians independent from the research team using SPSS software version 19.0 (SPSS, Chicago, IL, USA). | ______11_______ |
|  | 20b | Data analysis of baseline characteristics and of primary and secondary outcomes will be based on the intention-to-treat principle. χ2 Test will be conducted for the case of proportions and independent sample t tests will be analysed for testing the baseline differences between the two groups. A repeated-measures analysis of variance (ANOVA) will be used to compare the effect of two groups. Missing data will be replaced according to the principle of the last observation carried forward. | ______11______ |
|  | 20c | Statistical analyses will be performed on an intent-to-treat basis with missing data replaced by last observation values. | ______11_______ |
| **Methods: Monitoring** | | |  |
| Data monitoring | 21a | This trial will be monitored by the scientific research department of Beijing TCM Hospital. | ______11_____ |
|  | 21b | Description of any interim analyses and stopping guidelines, including who will have access to these interim results and make the final decision to terminate the trial | ______X______ |
| Harms | 22 | Any adverse events (described as unfavourable or unintended signs, symptoms or diseases occurring after treatment) related to acupuncture treatment will be observed and reported by patients and practitioners during each patient visit. In addition, all vital signs and adverse events will be measured and recorded at each visit. | ______10_____ |
| Auditing | 23 | The study will be audited by Beijing Municipal Science & Technology Commission of China every year. | ______X______ |
| Ethics and dissemination | | |  |
| Research ethics approval | 24 | The study protocol has been approved by the Research Ethics Committee of Beijing Traditional Chinese Medicine Hospital Affiliated to Capital Medical University (ref: 2014BL-063-02). | ____5,15__ _ |
| Protocol amendments | 25 | If it is nessrary to modify the protocol, we should submit applications to Beijing Municipal Science & Technology Commission of China. | _______X______ |
| Consent or assent | 26a | Researchers from Otorhinolaryngology Department, Peking University People’s Hospital will obtain informed consent or assent from potential trial participants. Participants will write informed consent. | _______5______ |
|  | 26b | Additional consent provisions for collection and use of participant data and biological specimens in ancillary studies, if applicable | ______X_______ |
| Confidentiality | 27 | Research data will be gathered and saved. Paper files will be kept in a locked filing cabinet. Electronic documents will be stored in a password protected computer, with access restricted to the principal investigator. All research documents will be preserved for at least 5 years after publication. | _____10________ |
| Declaration of interests | 28 | None | ______X_______ |
| Access to data | 29 | Dataset will be stored in a password protected computer, with access restricted to the principal investigator | ______10_______ |
| Ancillary and post-trial care | 30 | Provisions, if any, for ancillary and post-trial care, and for compensation to those who suffer harm from trial participation | ______X_______ |
| Dissemination policy | 31a | The results will be published after the study. | ______X_______ |
|  | 31b | Researcers in this trial will have authorship eligibility. | ______X_______ |
|  | 31c | The results will be published. | ______X_______ |
| Appendices |  |  |  |
| Informed consent materials | 32 | Model consent form and other related documentation given to participants and authorised surrogates | ______X_______ |
| Biological specimens | 33 | Plans for collection, laboratory evaluation, and storage of biological specimens for genetic or molecular analysis in the current trial and for future use in ancillary studies, if applicable | ______X_______ |

*It is strongly recommended that this checklist be read in conjunction with the SPIRIT 2013 Explanation & Elaboration for important clarification on the items. Amendments to the protocol should be tracked and dated. The SPIRIT checklist is copyrighted by the SPIRIT Group under the Creative Commons “[Attribution-NonCommercial-NoDerivs 3.0 Unported](http://www.creativecommons.org/licenses/by-nc-nd/3.0/)” license.
